# Lipid dysmetabolism in ceruloplasmin‐deficient mice revealed both *in vivo* and *ex vivo* by MRI, MRS and NMR analyses

**DOI:** 10.1002/2211-5463.13740

**Published:** 2023-12-15

**Authors:** Valeria Mannella, Linda Chaabane, Tamara Canu, Alan Zanardi, Sara Raia, Antonio Conti, Barbara Ferrini, Andrea Caricasole, Giovanna Musco, Massimo Alessio

**Affiliations:** ^1^ COSR‐Centre for Omics Sciences IRCCS‐San Raffaele Hospital Milano Italy; ^2^ Preclinical Imaging, Experimental Imaging Centre IRCCS‐San Raffaele Hospital Milano Italy; ^3^ Proteome Biochemistry, COSR‐Centre for Omics Sciences IRCCS‐San Raffaele Hospital Milano Italy; ^4^ Department of Research & Innovation, Kedrion S.p.A. Loc Bolognana Gallicano Italy; ^5^ Biomolecular Nuclear Magnetic Resonance, Division of Genetics and Cell Biology IRCCS‐San Raffaele Hospital Milano Italy; ^6^ Present address: LC, Euro‐BioImaging ERIC, Med‐Hub section, Institute of Biostructures and Bioimaging (IBB) Italian National Research Council (CNR) Torino Italy; ^7^ Present address: SR, Deloitte & Touche SpA Milano Italy

**Keywords:** aceruloplasminemia, ceruloplasmin, magnetic resonance imaging, magnetic resonance spectroscopy, nuclear magnetic resonance, steatosis

## Abstract

Ceruloplasmin (Cp) is a ferroxidase that plays a role in cellular iron homeostasis and is mainly expressed in the liver and secreted into the blood. Cp is also produced by adipose tissue, which releases it as an adipokine. Although a dysfunctional interaction of iron with the metabolism of lipids has been associated with several metabolic diseases, the role of Cp in adipose tissue metabolism and in the interplay between hepatocytes and adipocytes has been poorly investigated. We previously found that Cp‐deficient (CpKO) mice become overweight and demonstrate adipose tissue accumulation together with liver steatosis during aging, suggestive of lipid dysmetabolism. In the present study, we investigated the lipid alterations which occur during aging in adipose tissue and liver of CpKO and wild‐type mice both *in vivo* and *ex vivo*. During aging of CpKO mice, we observed adipose tissue accumulation and liver lipid deposition, both of which are associated with macrophage infiltration. Liver lipid deposition was characterized by accumulation of triglycerides, fatty acids and ω‐3 fatty acids, as well as by a switch from unsaturated to saturated fatty acids, which is characteristic of lipid storage. Liver steatosis was preceded by iron deposition and macrophage infiltration, and this was observed to be already occurring in younger CpKO mice. The accumulation of ω‐3 fatty acids, which can only be acquired through diet, was associated with body weight increase in CpKO mice despite food intake being equal to that of wild‐type mice, thus underlining the alterations in lipid metabolism/catabolism in Cp‐deficient animals.

## Graphical abstract

During aging, ceruloplasmin‐deficient (CpKO) mice display adipose tissue accumulation and liver steatosis. We investigated, both *in vivo* and *ex vivo*, the features of lipid dysmetabolism promoted by iron deposition in liver as a consequence of a deficiency of ceruloplasmin, a ferroxidase necessary for iron homeostasis. Our data revealed that iron deposition in liver fosters tissue inflammation and, subsequentially, lipid deposition and adipose tissue accumulation.

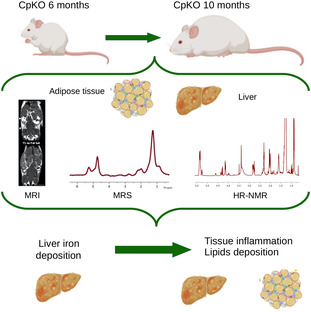

**Abbreviations**
ACp: aceruloplasminemia
ALT: alanine aminotransferase
AST: aspartate aminotransferase
AT: adipose tissue
CDCl_3_
: deuterated chloroform
Cp: ceruloplasmin
CpKO: ceruloplasmin knockout
FA: fatty acids
FWHM: frequency width at half magnitude
GTT: glucose tolerance test
HLC: hepatic lipids content
^1^H‐MRS: ^1^H‐magnetic resonance spectroscopy
^1^H‐HR‐NMR: ^1^H high resolution nuclear magnetic resonance
IHLC: intra‐hepatic lipid content
ITT: insulin tolerance test
LPC: lysophosphocholine
MCL: mean chain length
MRI: magnetic resonance imaging
MUFA: mono‐unsaturated fatty acids
NAFLD: non‐alcoholic fatty liver disease
ω‐3 fas: omega‐3 fatty acids
ndb: number of double bonds
PC: phosphocholine
PCA: principal component analysis
PE: phosphatidylethanolamine
PUFA: polyunsaturated fatty acids
RARE: relaxation enhancement
SFA: saturated fatty acids
SI: saturation index
TC: total cholesterol
TG: triglycerides
UFA: unsaturated fatty acids
WT: wild‐type

## Introduction

Ceruloplasmin (Cp) is a multi‐copper enzyme that plays a major role as plasma ferroxidase. However, several other less defined functions have been ascribed to it, including nitric oxide‐ and amine‐oxidase, copper transport, and anti‐oxidant and anti‐inflammatory activities [1, 2, 3, 4, 5]. According to its function, Cp plays a role in cellular iron homeostasis by promoting iron efflux, through the stabilization of the membrane iron exporter ferroportin, as well as the load of the oxidized form of iron onto transferrin [1, 6, 7, 8, 9]. *CP* gene deficiency, which results in a plasma ferroxidase activity defect, negatively impacts transferrin saturation, and causes iron‐restricted erythropoiesis, as well as iron accumulation in liver, pancreas, retina and brain [10]. In the rare genetic disease aceruloplasminemia (ACp) and in the Cp‐deficient (CpKO) mouse model, the absence or inactive form of the protein is associated with microcytic anemia and complications as a result of iron deposition, such as retinal damage, diabetes and neurodegeneration [8, 9, 10, 11, 12].

Cp is expressed as a secreted form and/or glycosylphosphatidylinositol‐membrane anchored isoform, in different cell type and tissues, *inter alia* the liver (the key tissue for iron homeostasis regulation, which secretes the protein into the blood) and the adipose tissue (AT), which releases it as adipokine [1, 7, 13, 14, 15]. Although altered interaction of iron and lipid metabolisms has been reported to characterize several metabolic diseases [16, 17, 18, 19], the role of Cp in AT metabolism and in the interplay between hepatocytes and adipocytes has been poorly investigated. In the absence of Cp activity in the liver and even in the presence of large iron deposition, no evident clinical features have been reported in addition to a generic hepatic inflammation in CpKO mice and liver steatosis in some ACp patients [15, 20, 21, 22]. In Cp‐deficient mice, we found that, during aging, the animals become overweight, showing AT accumulation and liver steatosis [23], suggestive of lipid metabolism dysregulation. Interestingly, AT of aged CpKO mice did not show alterations in iron homeostasis [23].

Using magnetic resonance imaging (MRI), ^1^H‐magnetic resonance spectroscopy (^1^H‐MRS) and high resolution nuclear magnetic resonance (^1^H‐HR‐NMR) techniques, we investigated the lipid alteration occurring during aging in the AT and liver of CpKO mice. We found AT accumulation both at 6 and 10 months of age in CpKO mice compared to wild‐type (WT). At 10 months of age lipid composition of AT was similar in the two groups, but CpKO mice showed adipocyte hypertrophy and macrophage infiltration, being the latter a sign of tissue inflammation. In the liver, along aging from 6 to 10 months, we observed accumulation of triglycerides and ω‐3 fatty acids (ω‐3 fas), through a switch from unsaturated to saturated fatty acids, which is characteristic of a transition from lipid utilization to lipid storage. The accumulation of ω‐3 fas, which can only be acquired with the diet because they are not synthesized by mammals, was associated with a CpKO genotype‐specific body weight increase, with food intake being equal to that of WT mice. Liver inflammation, as inferred by macrophage infiltration, was already occurring in CpKO mice at 6 months of age, in concomitance with large iron deposition. In 10‐month‐old CpKO mice, these features were associated with steatosis and hepatic damage. Consistent with findings in ACp patients [10, 21, 22, 24], these results underscore the altered lipid metabolism/catabolism in Cp deficiency and further support CpKO mice as a translationally relevant model for human ACp.

## Materials and methods

### Mouse model

CpKO mice, both males and females, from the original strain (C57Bl/6J genetic background) were used [12], whereas age‐ and sex‐matched WT C57Bl/6J mice were used as a control. The study was approved by the Institutional Animal Care and Use Committee (IACUC ID 1022, IRCCS‐OSR) and by the National Ministry of Health (n°77/2020‐PR). CpKO mice and WT mice (*n* = 5, three females and two males, each group) were evaluated at 6 and 10 months of age for body weight and *in vivo* analyzed by MRI and MRS. Blood was collected at 10 months of age at the end of the *in vivo* analyses, then mice were killed by transcardial perfusion with saline under deep anesthesia. Subsequently organs were collected and specimens were snap frozen or fixed for subsequent analysis.

On different groups of both CpKO and WT mice of 6 and 10 months of age (6 months bis and 10 months bis) (*n* = 6, three females and three males, each group) we performed the *in vivo* analysis of glucose tolerance test (GTT), insulin tolerance test (ITT) and food intake under a dark/light photocycle. The groups of mice at 6 months of age (6 months bis) were further used to collect organs and investigate pathological conditions of younger animals.

### *In vivo* MRI and MRS analysis

Two MRI exams were performed on each mouse at 6 and 10 months of age. All MRI scans were performed with a 7‐Tesla preclinical scanner (BioSpec 70/30 USR, Paravision 6.0.1; Bruker BioSpin MRI GmbH, Ettlingen, Germany) equipped with a circularly polarized volume coil with an inner diameter of 40 mm for spectroscopy or 70 mm for whole body imaging. All examinations were performed with mice under gas anesthesia (Isoflurane, 3% for induction and 1.5–2% for maintenance, mixed with oxygen) with breathing rate continuously monitored to adjust the anesthetic dose and with mice placed on a support with temperature control system to prevent hypothermia.

For the quantification of the AT volume, serial axial 2D‐high‐resolution rapid acquisition with relaxation enhancement (RARE) T2 images (52–62 axial slices, 1 mm thick) were acquired with or without fat suppression using the following sequence parameters: repetition time = 5600 ms; echo time = 40 ms; rare factors = 8: field of view = 35–38 × 22 mm; pixel = 0.137 × 0.137 mm; average of 5 scans. All axial sections were positioned to cover the entire abdominal area using sagittal and coronal T1‐weighted RARE images as anatomical references. The fat volume was determined by the segmentation of the subtracted fat suppression from without fat suppression images using mipav, version 8.0.2 (National Institutes of Health, Bethesda, MA, USA). Multispectral fuzzy C‐means clustering algorithm was applied on the resulting whole images to perform ‘hard and fuzzy segmentation’. AT volume quantification was calculated using the Paint Grow tool on each slice and was normalized for the animal length, which was measured on RARE‐T1 sagittal images. For liver volume quantification, manual liver segmentation was performed on each slice using the RARE‐T2 sequence without fat suppression and the liver volume results from automatic summation of voxel volumes; images were analyzed with mipav.

Proton magnetic resonance spectroscopy (^1^H‐MRS) was performed to evaluate the intrahepatic lipid content (IHLC). Axial, coronal and sagittal T2 weighted MRI images were used to define the overall liver volume and place a voxel (3 × 3 × 3 mm^3^) of interest for ^1^H‐MRS. MR spectra were acquired with the point‐resolved spectroscopy sequence with or without water suppression (repetition time = 2500 ms; echo time = 16.6 ms; spectral width = 4504.50 Hz; 2048 points; average of 12 scans), reconstructed with topspin (PV6.0; Bruker BioSpin). Automatic quantification of *in vivo* ^1^H‐MR spectra was performed with lcmodel (http://lcmodel.com/lcmodel.shtml) [25]. *In vivo* evaluation of liver fatty acids contents and composition was performed in mice at 6 and 10 months. IHLC quantitation was calculated as the ratio of (‐CH_2_‐)_n_/water peak integral areas of water‐unsuppressed spectra as described previously [26, 27]. The intrahepatic fatty acids (FA) composition indices were calculated as described previously [27, 28] using the integral area of peaks of resonance of specific chemical groups of the water‐suppressed spectra. The calculated indices were: saturation index (SI), number of double bonds (ndb), unsaturated fatty acids (UFA), saturated‐FA (SFA), polyunsaturated‐FA (PUFA), monounsaturated fatty acids (MUFA) and mean chain length (MCL) (Table S1). Triglycerides (TG) content was calculated according to: $\frac{A_{4.3\mathrm{ppm}}+A_{4.1\mathrm{ppm}}}{4}$ where *A*_4.3ppm_ and *A*_4.1ppm_ are the integral area of the peaks at 4.3 ppm and 4.1 ppm chemical shift, respectively, and 4 is the number of protons present in the functional group defining the TG at the quoted peaks.

### ^1^H‐HR‐NMR analysis

Snap‐frozen samples of liver and perigonadal AT were weighed before the analysis (fresh weight) and in the case of liver also after 24 h lyophilization (dry weight). Polar and apolar metabolites were extracted from either fresh tissue or dry tissue powder using a MeOH/CHCl_3_/H_2_O (2/2/1: v/v/v) solvent extraction strategy. Apolar phases were lyophilized for 24 h and the resulting powders were then suspended in 250 μL of deuterated chloroform (CDCl_3_). ^1^H‐HR‐NMR spectra were recorded at 298 K on a Bruker Avance 600 Ultra Shield TM Plus 600 MHz spectrometer equipped with triple resonance cryoprobe, pulsed field gradients. 1D ^1^H‐HR‐NMR spectra (noesy1d) were recorded with an acquisition time of 6.8 s, 128 transients and a relaxation delay of 6 s. The spectral window was set to 12 ppm. 1D ^1^H‐HR‐NMR spectra were typically processed with zero filling to 132 k points, and apodized with an unshifted Gaussian and a 1 Hz line broadening exponential using mestrenova 14.0 (Mestrelab Research, Santiago, Spain). Peaks assignment was performed in accordance with the literature [29, 30, 31] and taking advantage of our in‐house database (see below and Table S2). Spectra were referenced to the chloroform signal at δ = 7.26 and manually integrated using the ‘peaks’ method in mestrenova 14.0. NMR data were then normalized to total area, Pareto scaled and subjected to principal component analysis (PCA) using r, version 4.0.2 (R Foundation, Vienna, Austria) and rstudio, version 1.3.1073 (RStudio, Boston, MA, USA). Total lipid content was evaluated for each sample as total area of NMR spectrum and then normalized to the dry weight for liver samples and for the fresh weight for perigonadal AT. To obtain absolute lipids quantification, an in‐house database was built using 21 commercial molecules (Merck, Darmstadt, Germany; Sigma‐Aldrich, St Louis, MO, USA) belonging to 11 lipids classes (Table S2). Powders were re‐constituted in 100% CDCl_3_ at known concentration (i.e. 10 mm for all molecules except for TG, 36.7 mm) and 250 μL of volume was transferred in a 3‐mm diameter glass NMR tube. 1D ^1^H‐HR‐NMR spectra were acquired as described above.

The lipids concentration was calculated using [32]:

$${\text{Conc}}_{\mathrm{met}}=\frac{{\text{Conc}}_{\mathrm{st}}*A_{\mathrm{met}}*m}{A_{\mathrm{st}}*m_{\mathrm{st}}}$$

$$m=\frac{T*P1}{\mathrm{RG}*\mathrm{NS}}$$

where *Conc*_*met*_ and *Conc*_*st*_ are the concentration of the specific metabolite in the sample and in the corresponding standard, respectively; *A*_*met*_ and *A*_*st*_ are the integrals of the specific metabolite and standard, respectively; and *m* and *m*_*st*_ are the acquisition parameters [temperature (T), number of scans (NS), P1 pulse at 90° and Receiver gain (RG)] of 1D ^1^H‐HR‐NMR spectra acquired on sample and reference spectra.

### Total iron evaluation by the ferene‐S assay

The assay is modified from Riemer *et al*. [33] using ferene‐S [3‐(2‐pyridyl)‐5,6‐di(2‐furyl)‐1,2,4‐triazine‐5′,5′′‐disulfonic acid disodium salt] (Merck 82940). Specimens of liver tissue were solubilized in 50 mm NaOH (2 h at 37 °C) and mixed (1 : 1) with 10 mm HCl. To this mixture, the iron releasing reagent (a 1 : 1 solution of 4.5% KMnO_4_ and 1.4 m HCl, freshly prepared) was added (1 : 2) and samples were incubated 2 h at 60 °C under shaking. After cooling to room temperature, the detecting reagent (1 m ascorbate, 2.5 m ammonium acetate, 130 mm thiourea and 6.5 mm ferene‐S) was added (1 : 10) to the mixture and samples were incubated (30 min at 20 °C) under shaking. Absorbance was measured in triplicate, 100 μL per well in 96‐well plates, at 595 nm with microplate reader (iMark; Bio‐Rad, Hercules, CA, USA). Protein concentration was determined by Bradford assay in the original lysate solubilized in NaOH and used to normalize iron with protein content.

### Histological and immunohistological analysis

Liver and adipose tissues were fixed in 4% paraformaldehyde (1 h at 4 °C), transferred into 70% ethanol solution and embedded in paraffin 24 h later. Hematoxylin and eosin staining and immunostaining for F4/80 antigen detection were performed on a 3‐μm thick section (Animal Histopathology Facility, San Raffaele Hospital). Automated immunostaining for macrophage marker F4/80 and cell counterstaining was performed with F4/80 (D2S9R) XP® Rabbit monoclonal antibody (CST#70076; Cell Signaling Technology, Danvers, MA, USA) and hematoxylin, respectively, using BOND RX instrumentation (Leica, Wetzlar, Germany). Samples were analyzed with an AxioImager microscope (Zeiss, Oberkochen, Germamy) and images analysis was performed using qupath (https://github.com/qupath) [34]. Lipid macrovesicle and F4/80 positive cells areas in liver were quantified on four images from two different tissue sections acquired for each mouse and data are reported as a percentage of total pixel area. F4/80 positive cells in the AT were reported as a percentage of total cells automatically detected by qupath, whereas the size of adipocytes was quantified on the histological images for circular area and diameter detection. Four images per mouse were acquired and data are reported as pixel area and diameter pixel length.

### TG evaluation

Liver TG were determined using the EnzyChrome triglyceride assay kit (BioAssay Systems, Hayward, CA, USA). Tissue (10 mg) were homogenized in 100 μL of 5% Triton X‐100 and incubated in water bath for 5 min in which the temperature increases from 80 to 100 °C. The procedure was repeated three times, allowing the samples to settle at 20 °C between cycles. Samples were then centrifuged (16 000 ***g*** for 5 min at 4 °C), the supernatant was diluted 1 : 10 in MilliQ water (Millipore, Burlington, MA, USA) and 10 μL of this was used for the assay performed in triplicate in 96‐well plates. Samples were mixed with 100 μL of kit working reagent and incubated (15 min at 20 °C) before plates were read at 595 nm wavelength in a Bio‐Rad iMark microplate spectrophotometer.

### GTT and ITT

For GTT, mice were fasted 16 h overnight and blood glucose level was measured using a Contour Care glucometer (Ascensia Diabetes Care, Basel, Switzerland) at baseline (time 0) and then at 15, 30, 60 and 120 min after intraperitoneal injection of glucose (2 mg·g^−1^ mouse body weight). ITT was performed after a 4 h fast measuring blood glucose at baseline and at 15, 30, 60, 90 and 120 min after intraperitoneal injection of insuline (0.75 mU·g^−1^ mouse body weight).

### Biochemical parameters in the serum

Blood collected from mice at 6 and 10 months of age was analyzed for iron, alanine aminotransferase (ALT) and aspartate aminotransferase (AST) concentrations in the serum at the San Raffaele Mouse Clinic‐Animal Biochemistry facility using the ILab 650 clinical chemistry system (Instrumentation Laboratory, Befrord, MA, USA) for analyses of biochemistry.

### Food intake

In different groups of mice (*n* = 6, three males and three females both for CpKO and WT), the evaluation of food intake was performed every months from 4 to 10 months of age of the animals by replacing sawdust with a sheet of 3‐mm paper and providing 30 g of food pellet within a 6‐cm diameter dish without a lid. Food intake was measured for a total of 3 days. Dishes with the food were weighed at the beginning and the end of the 3 days. Food intake was measured as the difference in weight between the food provided and that remaining in the cage at the end of the 3 days, taking crumbs into account.

To evaluate possible differences of food intake in light and dark cycles in the selected groups of mice (animals at 6 and 10 months of age; *n* = 6, three males and three females), food intake was measured every 12 h during the light and dark cycles for a total of 3 days, as described above. Food intake was measured as the difference in weight between the food put into the cage and that remaining at the end of the dark or light cycle, taking crumbs into account.

### Statistical and bioinformatics analysis

Data from two different groups were evaluated using an unpaired Student's *t* test if they passed the normality test for Gaussian distribution (Kolmogorov–Smirnov test) or they were evaluated by Mann–Whitney test. In all analyses *P* < 0.05 (two‐tailed) was considered statistically significant comparing the mean ± SEM. Correlation analysis was performed using either Pearson's test or Sperman's test. Analyses were performed with either prism, version 9.0 (GraphPad Software Inc., San Diego, CA, USA) or r, version 4.0.2, and rstudio, version 1.3.1073.

## Results

### CpKO mice are overweight compared to WT and show AT accumulation

**Fig. 1:**
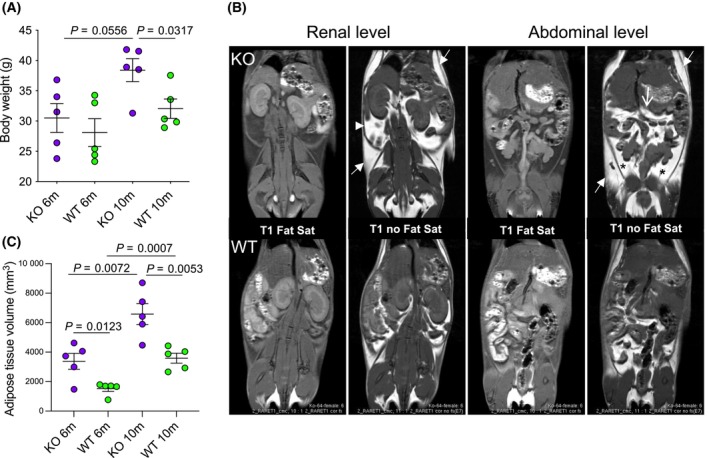
CpKO mice are overweight compared to WT and show AT accumulation. (A) Body weight of mice at 6 and 10 months of age. (B) Representative sagittal 2D‐high‐resolution rapid acquisition with relaxation enhancement T1 MRI images acquired with (Fat Sat) or without fat suppression (no Fat Sat) at renal and abdominal levels, used for *in vivo* quantification of the AT volume in CpKO and WT mice as indicated in the Materials and Methods. Arrows indicate subcutaneous AT, head‐arrow indicates perirenal AT, open arrow indicates visceral AT and asterisks indicate perigonadal AT. For images of all mice included in the study, see Fig. S1. (C) Quantitation of MRI‐determined AT volume of CpKO and WT mice at 6 and 10 months of age normalized for the animal length. Data are the mean ± SEM of animal groups, with each dot corresponding to one animal (*n* = 5 per group); *P* values were evaluated by Mann–Whitney's test or Student's *t* test in (A) and (C), respectively.

We examined CpKO mice starting from an age of 6 months, at the onset of a detectable neurological impairment being first apparent [23, 35]. At 6 months of age CpKO and WT mice showed comparable body weight, whereas, at 10 months, they were significantly overweight compared to WT mice (Fig. 1A). *In vivo* MRI of fat‐saturated and fat‐non saturated images allowed detection of AT accumulation in CpKO mice at perirenal, visceral, subcutaneous and perigonadal regions (Fig. 1B). The quantification of AT volume, normalized for animal length, showed higher AT accumulation in CpKO mice than in WT mice both at 6 and 10 months, being more significant at 10 months (Fig. 1C; Fig. S1). A significant AT accumulation during aging was observed for both CpKO and WT mice. These data suggested that lipid dysmetabolism was already occurring in CpKO mice at 6 months of age.

### CpKO mice show adipocyte hypertrophy and macrophage infiltration in the AT

**Fig. 2:**
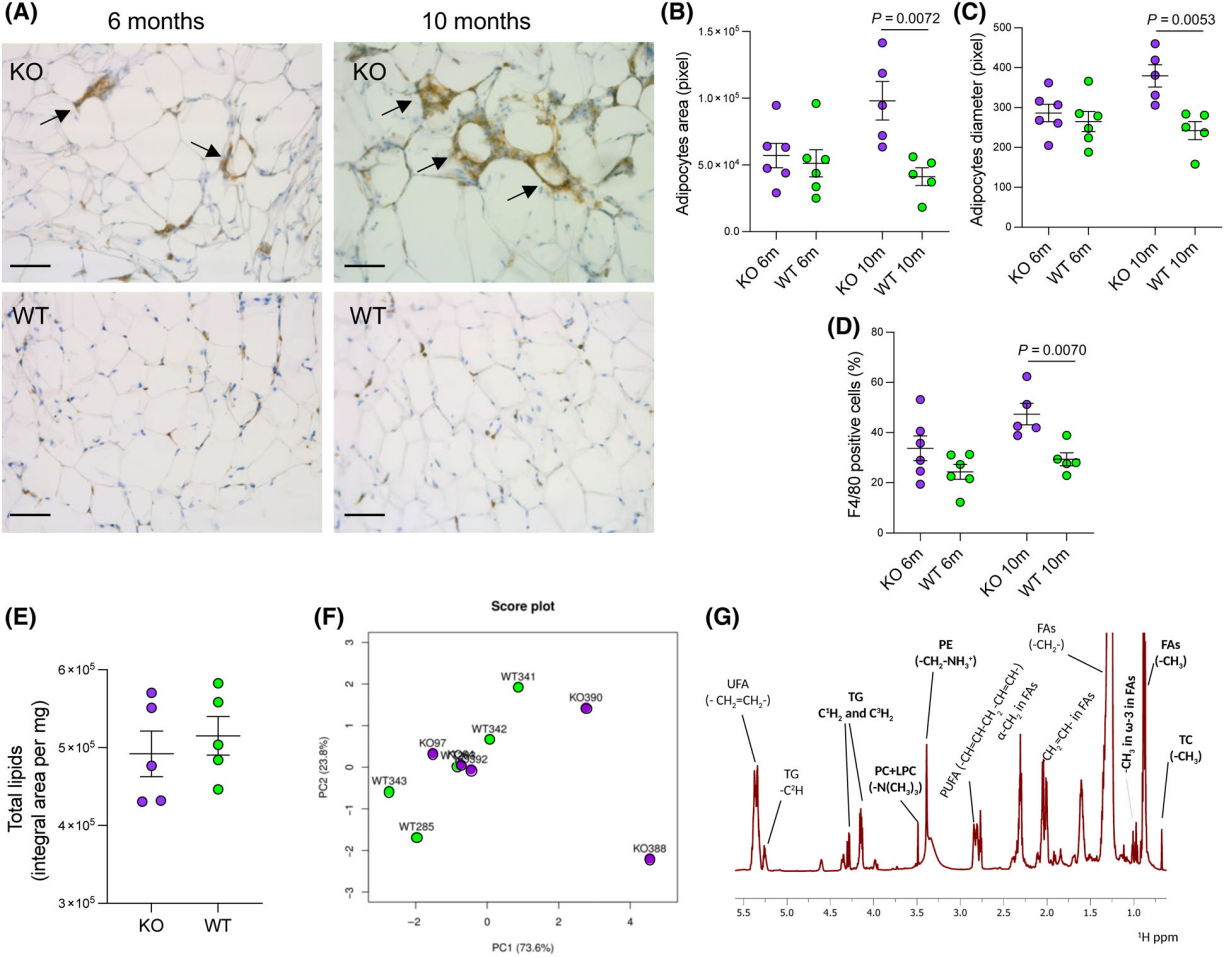
CpKO mice at 10 months show adipocyte hypertrophy and macrophage infiltration in the AT, which exhibits a lipid composition similar to WT mice. (A) Representative immunohistological sections of perigonadal AT stained for the F4/80 macrophages marker (brown) and counterstained with hematoxylin (blue). Arrows indicate clusters of infiltrating macrophages. Images were homogenously corrected for brightness. Scale bars = 100 μm. (B, C) Analysis of adipocytes size reported as an average of pixel area (B) and pixel diameters (C). (D) Quantitation of F4/80 staining reported as a percentage of positive cells as detected with QuPath software. (E) Quantitation of total lipids reported as integral area, normalized by milligrams of fresh tissue, obtained from full ^1^H‐HR‐NMR spectrum of AT apolar extracts of CpKO and WT mice at 10 months of age. Data are the mean ± SEM of animal groups, with each dot corresponding to one animal (*n* = 6 per group and *n* = 5 per group for animals at 6 and 10 months, respectively); *P* values were evaluated by Student's *t* test. (F) Score plot of PCA of the 1D ^1^H‐HR‐NMR spectra profiles. Each dot corresponds to one mouse (CpKO, purple; WT, green) distributed upon dimensional reduction according to the first two principal components (PC1 and PC2). (G) Lipidic profile of the AT. Representative 1D ^1^H‐HR‐NMR spectrum acquired on the apolar part of the AT. The different chemical groups are identified according to the specific resonance of the proton in the fatty‐acyl chains. Functional groups in bold were quantified as absolute millimolar concentration because it was possible to determine the number of protons contributing to the specific signal. The other lipids functional groups were identified and quantified as relative abundance, measured as ratio of specific peak area/total peaks area. Lipid classes defined by the functional groups were also indicated: ω‐3 fas, omega‐3 fatty acids; FAs, fatty acids; MUFA, mono‐unsaturated fatty acids; PC + LPC, phosphocholine and lysophosphocholine; PE, phosphatidylethanolamine; PUFA, polyunsaturated fatty acids; TC, total cholesterol; TG, triglycerides; UFA, unsaturated fatty acids.

We then evaluated in the AT adipocytes size, as a sign of hypertrophy, and macrophage infiltration, as indicator of tissue inflammation. CpKO mice showed significant adipocyte hypertrophy at 10 months of age compared to WT mice, as inferred from larger size area and diameter, which was not present in younger mice (Fig. 2A–C).

A trend for an increase in macrophage infiltration in the AT of CpKO mice was observed at 6 months of age, and the number of infiltrating cells was significantly higher in the CpKO mice compared to WT mice at 10 months of age (Fig. 2A,D). Interestingly, both at 6 and 10 months of age, in the CpKO mice, the staining of F4/80 marker of macrophages highlighted clusters of cells around vessels and adipocytes (Fig. 2A, arrows), whereas, in WT mice, the staining was dispersed as single cells. This was reminiscent of a diffuse immune‐surveillance in the WT mice and of an active infiltration as a result of inflammation in CpKO mice. These analyses were performed in the animals at 10 months of age at the end of the MRI longitudinal analysis and in new groups of 6‐month‐old mice.

### Lipid composition of the AT of CpKO mice is similar to WT mice

An ^1^H‐HR‐NMR analysis of the lipid composition of the perigonadal AT was performed *ex vivo* on the specimens collected from 10‐month‐old mice. Despite adipocyte hypertrophy and larger macrophage infiltration, the CpKO mice did not show gross alteration in the lipid composition of the AT. Indeed, total lipid content was comparable between CpKO and WT (Fig. 2E). Accordingly, PCA performed on the profiles of the lipid full spectra was unable to distinguish the two groups of mice (Fig. 2F). These results suggested a similar lipid composition of the AT in the two groups of animals.

Functional chemical groups belonging to lipids, and some of the lipid classes attributable to them, were identified and quantified comparing the spectra of standard lipids after normalization according to the tissue fresh weight of each sample (Fig. 2G). The results indicated comparable concentration of TG, total cholesterol (TC), FA and ω‐3 fas in the AT of CpKO and WT mice (Table 1 and Table S3 for the value of each mouse). Of interest was the detection of ω‐3 fas because these lipids cannot be synthesized by mammals and are only derived from the diet.

**Table 1:** ^1^H resonance assignments and quantification of the lipid classes by NMR in the adipose tissue of 10 months mice. ω‐3 Fas, omega‐3 fatty acids; FA, fatty acids; FC, fold changes; FW, fresh weight; LPC, lysophosphocholine; NS, not significant; PC, phosphocholine; PE, phosphatidylethanolamine; TC, total cholesterol; TG, triglycerides. *P* value: Student's *t* test. A bold **H** indicates the signal selected for quantification.

Peaks (chemical shift ppm)	Functional group	Lipid class	CpKO		WT		FC KO/WT	*P* value
			mm·mg^−1^ FW	SD	mm·mg^−1^ FW	SD		
4.30–4.12	(‐C^1^ **H**_2_);(‐C^3^ **H**_2_)	TG	0.7776	0.1008	0.8148	0.0894	0.9543	NS
3.48	(‐N(C**H**_3_)_3_)	PC + LPC	0.0012	0.0006	0.0008	0.0003	1.4844	NS
3.4	(‐C**H**_2_‐NH_3_^+^)	PE	0.0019	0.0024	0.0042	0.0024	0.4564	NS
0.98	(‐C**H**_3_)	ω‐3 Fas	0.0543	0.0111	0.0563	0.0108	0.9646	NS
0.9	(‐C**H**_3_)	FA	1.7879	0.2284	1.8749	0.2090	0.9536	NS
0.68	(‐C**H**_3_)	TC	0.0026	0.0004	0.0026	0.0005	0.9962	NS

Other lipid functional chemical groups were not quantified absolutely because it was not possible to determine the number of protons contributing to their specific signals (Fig. 2G). The relative abundance of these functional groups measured as the ratio between the specific peak area over the total peaks area did not show significant differences between CpKO and WT mice (data not shown).

### CpKO mice at 6 months show higher intrahepatic TG content than WT and a switch from unsaturated to saturated fatty acids with aging

**Fig. 3:**
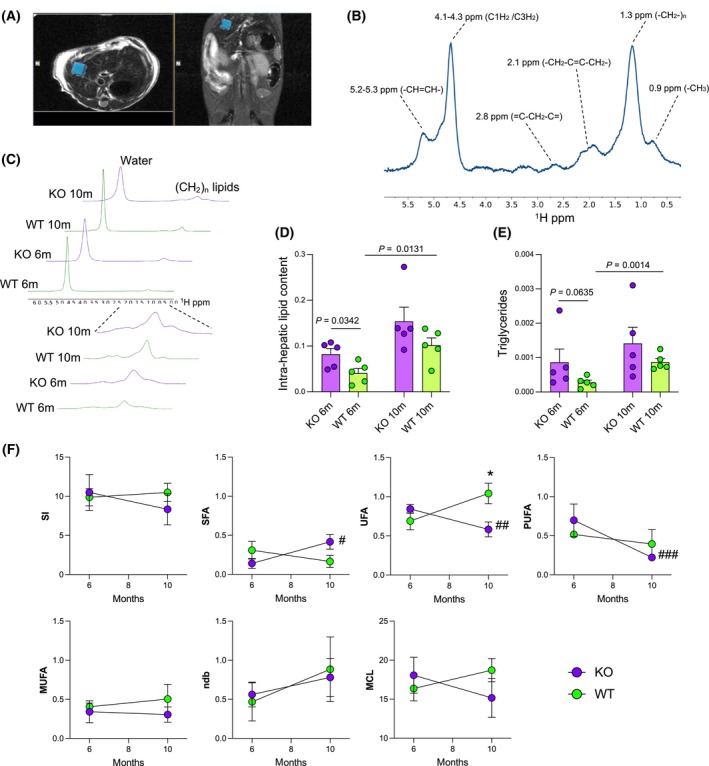
CpKO mice at 6 months of age show higher intrahepatic lipid and TG content than WT mice displaying a switch from unsaturated to saturated fatty acids with aging. (A) Representative T2 MRI images of mouse axial and coronal sections at the liver level where the voxel of interest (blue squares) was placed for ^1^H‐MRS acquisition. (B) Representative ^1^H‐MRS spectrum of the liver, acquired with water suppression, showing the different types of protons in the functional groups of FA, defined according to their chemical shifts. (C) Representative ^1^H‐MRS spectra without water suppression acquired *in vivo* on the liver of one CpKO and one WT mouse at 6 and 10 months showing water resonance at 4.7 ppm and main lipid component resonance at 1.3 ppm. The expanded part of the spectra shows the differences between CpKO and WT mice in the peak area used for intrahepatic lipid content quantification. (D) Intrahepatic lipid content evaluation reported as ratio of (‐CH_2_‐)_n_/water peak integral areas of water‐unsuppressed spectra. (E) Liver TG evaluation *in vivo* in mice at 6 and 10 months reported as (*A*_4.3ppm_ + *A*_4.1 ppm_)/4, where *A*_4.3ppm_ and *A*_4.1ppm_ are the integral area of the peaks at 4.3 and 4.1 ppm chemical shift and 4 is the number of protons present in the functional group defining the TG at the quoted peaks. Data are reported as the mean ± SEM of the mouse groups (purple, CpKO; green, WT); each dot corresponds to one animal (*n* = 5 per group); *P* values were evaluated by Student's *t* test in (D) or Mann–Whitney test in (E). (F) Evolution of the FA composition indices along aging from 6 to 10 months in CpKO and WT mice. MCL, mean chain length; MUFA, mono‐unsaturated fatty acids; ndb, number of double bonds; PUFA, polyunsaturated fatty acids; SFA, saturated fatty acids; SI, saturation index; UFA, unsaturated fatty acids. Data are reported as the mean ± SEM of the animal groups at the indicated age; statistical *P* values were evaluated by comparing the CpKO and WT groups at the same age (*P = 0.0225, Student's *t* test) or the same animal group at the different ages (^#^ *P* = 0.0422; ^##^ *P* = 0.0486, Student's *t* test; ^###^ *P* = 0.0079, Mann–Whitney test).

*In vivo* evaluation of liver fatty acids contents and composition was performed by MRS in mice at 6 and 10 months of age in a 3 × 3 × 3 mm^3^ voxel (Fig. 3A). The FA composition indices were calculated using the integral area of peaks of resonance of specific chemical groups of the water‐suppressed spectra as described previously [26, 27] (Fig. 3B and Table S1). At 6 months of age, IHLC calculated as the ratio of (‐CH_2_‐)_n_/water peak integral areas of water‐unsuppressed spectra showed a significant accumulation in CpKO compared to WT mice, whereas, at 10 months, the IHLC was not significantly different between the two groups of animals (Fig. 3C,D; Table 2 and Table S3 for the value of each mouse). Both CpKO and WT mice showed an increase in hepatic lipid accumulation between 6 and 10 months of age, which was significant in WT mice (Fig. 3C,D; Table 2 and Table S3). The TG content, which is indicative of lipid deposition/accumulation, behaved similarly to the IHLC, being slightly higher in CpKO than WT mice, and, in both group of animals, it showed an increased accumulation with aging (Fig. 3E and Table 2 and Table S3).

**Table 2:** Intrahepatic lipid content, triglycerides evaluation, and characterization of fatty‐acyl chains by ^1^H‐MRS *in vivo* in the liver of CpKO and WT mice at 6 and 10 months of age. a.u., arbitrary units; FC, fold changes; IHCL, intra‐ehapatic lipid content; MCL, mean chain length; MUFA, monounsaturated fatty acids; ndb, number of double bonds; NS, not significant; PUFA, polyunsaturated fatty acids; SFA, saturated fatty acids; SI, saturation index; TG, triglycerides; UFA, unsaturated faty acids. *P* values were evaluated by Student's *t* test or the Mann–Whitney test.

Indices	6 months						10 months						6 vs 10 months			
	CpKO		WT		FC KO/WT	*P* value	CpKO		WT		FC KO/WT	*P* value	CpKO		WT	
	a.u.	SD	a.u.	SD			a.u.	SD	a.u.	SD			FC 10/6	*P* value	FC 10/6	*P* value
IHLC	0.082	0.028	0.041	0.023	2.00	0.0342	0.154	0.07	0.102	0.036	1.51	NS	1.88	0.0645	2.49	0.0131
TG	0.00086	0.0009	0.0003	0.0002	3.07	0.0635	0.00141	0.0011	0.0009	0.0002	1.62	NS	1.64	NS	3.11	0.0014
SI	10.50	5.12	9.88	2.46	1.06	NS	8.34	4.46	10.50	2.62	0.79	NS	0.79	NS	1.06	NS
ndb	0.56	0.35	0.47	0.55	1.19	NS	0.78	0.54	0.88	0.93	0.89	NS	1.39	NS	1.87	NS
UFA	0.84	0.13	0.69	0.25	1.53	NS	0.58	0.21	1.04	0.29	0.56	0.0225	0.69	0.0486	1.51	NS
SFA	0.14	0.14	0.31	0.25	0.45	NS	0.42	0.21	0.17	0.17	2.47	0.0766	2.97	0.0422	0.54	NS
PUFA	0.70	0.46	0.52	0.08	1.35	NS	0.22	0.09	0.39	0.41	0.56	NS	0.31	0.0079	0.75	NS
MUFA	0.34	0.31	0.41	0.11	0.84	NS	0.31	0.22	0.5	0.42	0.61	NS	0.91	NS	1.22	NS
MCL	18.06	5.15	16.37	3.57	1.1	NS	15.16	5.60	18.71	3.34	0.81	NS	0.84	NS	1.14	NS

The indices characterizing the FA composition showed a significant reduction of UFA in CpKO compared to WT mice at 10 months of age. A significant reduction of both UFA and PUFA was also observed in CpKO mice at 10 months compared to the younger animals (Fig. 3F and Table 2). Interestingly, the FA saturation in CpKO and WT mice displayed an opposite trend during aging. Indeed, CpKO mice showed an increase in SFA, suggestive of incremented lipid deposition, which was the opposite in WT mice (Fig. 3F and Table 2).

Taken together these results indicated that CpKO mice already displayed lipid metabolism alterations in the liver at 6 months of age, showing higher lipids/TG deposition than WT mice, and, in aged CpKO mice, FA acquire features of lipid accumulation/storage characterized by an increase in saturation.

### The *ex vivo* analysis at 6 and 10 months of age shows liver steatosis and macrophage infiltration in CpKO mice, which results in liver damage

**Fig. 4:**
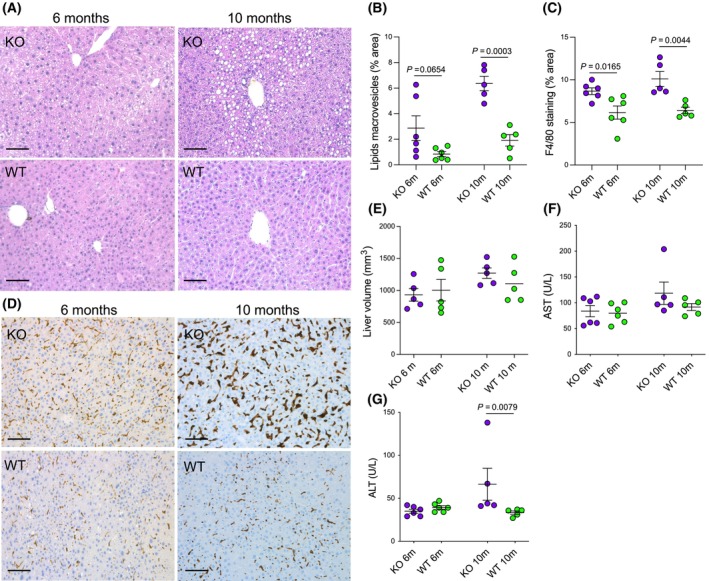
*Ex vivo* analysis at 6 and 10 months of age shows liver steatosis and macrophage infiltration in CpKO mice that results in liver damage. (A) Representative hematoxylin and eosin staining on histological liver sections from 6‐ and 10‐month‐old CpKO and WT mice. Scale bars = 100 μm. (B) Quantitation of lipids droplets determined as a percentage of area occupied by macrovesicles in liver sections. (C) Quantitation of F4/80 macrophages marker staining reported as a percentage of area occupied by brown staining in the liver section. (D) Representative immunohistological sections of liver stained for the F4/80 macrophages marker (brown) and counterstained with hematoxylin (blue). Scale bars = 100 μm. (E) Liver volume quantification based on the reconstruction of liver segmentation from MRI images. Volume is normalized for body weight. (F, G) Evaluation of circulating hepatic enzymes AST and ALT, respectively. Data are the mean ± SEM of animal groups, with each dot corresponding to one animal (*n* = 6 per group and *n* = 5 per group for animals at 6 and 10 months, respectively); *P* values were evaluated by Student's *t* test in (B), (C), (E) and (F) or Mann–Whitney's test in (G).

The indications on hepatic lipid content obtained by *in vivo* MRS analysis were confirmed by the *ex vivo* analysis on the same mice at 10 months and in a different group of 6‐month‐old mice. Indeed, histological analysis revealed a significantly more substantial presence of intracellular lipids macrovesicles in CpKO mice than in WT mice at 10 months (Fig. 4A,B), which reflected accumulation of lipid droplets and steatosis, as reported previously [23]. However, a trend for an increase in the accumulation of intracellular lipids was already observable at 6 months in CpKO mice, at least in some animals where lipid deposition was abundant (Fig. 4A,B). Accumulation of lipid droplets in CpKO mice was associated with liver inflammation, as inferred from a significant increase in liver macrophage infiltration, which was present at 6 months of age and increased in older mice (Fig. 4C,D). Despite accumulation of liver lipids, CpKO mice did not show overt hepatomegaly as indicated by both liver volume reconstruction on MRI images and visual inspection of the collected organs (Fig. 4E and Fig. S2). Accumulation of lipids in the liver of CpKO mice did not promote metabolic disorders such as glucose intolerance and/or insulin resistance either in 6‐month‐old mice or aged animals (Fig. S3). By contrast, in aged CpKO mice, liver steatosis and inflammation resulted in a significant increase in the circulating hepatic enzyme ALT indicating liver damage (Fig. 4F,G).

### Liver lipid deposition is characterized by accumulation of triglycerides, fatty acids and ω‐3 fatty acids

**Fig. 5:**
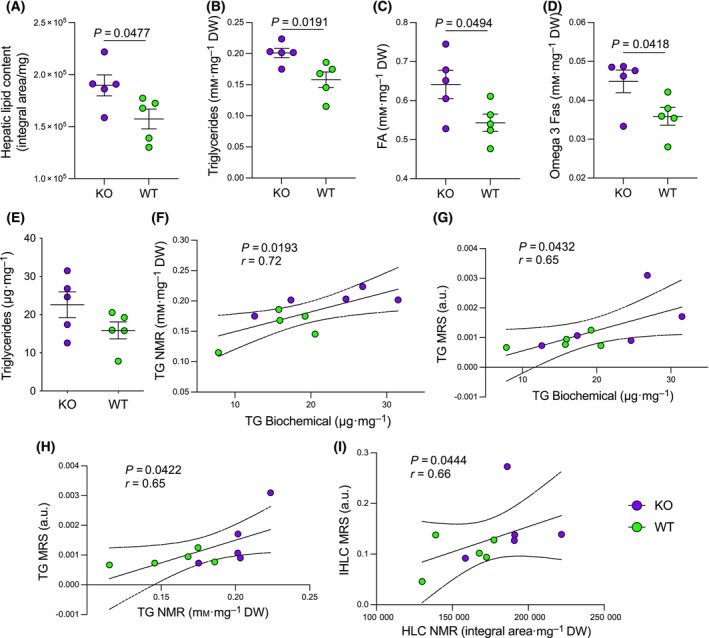
Liver lipid deposition is characterized by accumulation of triglycerides, fatty acids and ω‐3 fatty acids. (A–D) Quantitation from ^1^H‐HR‐NMR spectrum of liver extracts of CpKO and WT mice at 10 months of age of the (A) total hepatic lipids content reported as integral area pixel normalized by milligrams of dry weight tissue, (B) triglycerides, (C) fatty acids and (D) omega‐3 fatty acids reported as millimolar concentration by milligrams of dry weight tissue. Data are the mean ± SEM of animal groups, with each dot corresponding to one mouse (*n* = 5 per group); *P* values were evaluated by Student's *t* test. (E–I) Comparison of measurements by different methodologies for the assessment of TG and total lipid content. (E) TG quantitation by biochemical assay in the liver extract of CpKO and WT mice at 10 months of age. Data are the mean ± SEM of animal groups, with each dot corresponding to one mouse (*n* = 5 per group); *P* values were evaluated by Student's *t* test. (F) Correlation analysis of the TG evaluation obtained by biochemical assay versus ^1^H‐HR‐NMR or (G) versus MRS quantitation. (H) Correlation analysis of the TG evaluation obtained by ^1^H‐HR‐NMR versus MRS. (I) Correlation analysis of the total lipids content evaluation obtained by ^1^H‐HR‐NMR versus MRS. Each dot corresponds to one mouse; *P* values were evaluated by Pearson's test (F–H) or Spearman' test (I) and the linear correlation coefficient *r* is reported; dashed lines indicated the 95% confidence interval around the interpolation line.

^1^H‐HR‐NMR analysis performed on the liver collected from 10‐month‐old mice showed a significant higher amount of total hepatic lipids content (HLC) in CpKO mice compared to WT mice (Fig. 5A). Moreover, some specific chemical groups were assigned to specific lipid classes and quantified. The TG component mainly contributed to the HLC accumulation in CpKO mice, with higher concentrations compared to WT mice (Fig. 5B and Table 3). In addition to TG, the FA and the ω‐3 fas accumulated in CpKO and were significantly higher than in WT mice (Fig. 5C,D, and Table 3; see also Table S3 for the values of each mouse). We did not detect significant differences between CpKO and WT mice with respect to the absolute amount of the other lipids identified (i.e. PC + LPC, PE and TC). Similar to AT, the relative abundance of the other identified functional groups did not show significant differences between CpKO and WT mice (data not shown).

**Table 3:** ^1^H resonance assignments and quantification of the lipid classes by NMR in the liver of 10 months mice. ω‐3 Fas, omega‐3 fatty acids; FA, fatty acids; FC, fold changes; FW, fresh weight; LPC, lysophosphocholine; NS, not significant; PC, phosphocholine; PE, phosphatidylethanolamine; TC, total cholesterol; TG, triglycerides. *P* value: Student's *t* test. Bold **H** indicates the signal selected for quantification.

Peaks (chemical shift ppm)	Functional group	Lipid class	CpKO		WT		FC KO/WT	*P* value
			mm·mg^−1^ DW	SD	mm·mg^−1^ DW	SD		
4.30–4.12	(‐C^1^ **H**_2_);(‐C^3^ **H**_2_)	TG	0.2011	0.0172	0.1579	0.0281	1.273	0.0191
3.48	(‐N(C**H**_3_)_3_)	PC + LPC	0.0036	0.0022	0.0060	0.0064	0.596	NS
3.4	(‐C**H**_2_‐NH_3_^+^)	PE	0.0269	0.0043	0.0357	0.0176	0.756	NS
0.98	(‐C**H**_3_)	ω‐3 Fas	0.0449	0.0065	0.0359	0.0051	1.250	0.0418
0.9	(‐C**H**_3_)	FA	0.6416	0.0811	0.5433	0.0496	1.181	0.0494
0.68	(‐C**H**_3_)	TC	0.0186	0.0053	0.0167	0.0035	1.112	NS

Using a biochemical assay, we also evaluated the level of TG in the liver extracts of 10‐month‐old mice, which showed a trend towards an increase in CpKO mice compared to WT (Fig. 5E). The TG levels of CpKO and WT mice measured by the biochemical assay exhibited a significant correlation with the TG levels measured both by NMR and MRS (Fig. 5F,G), Similarly, we observed a good correlation between NMR and MRS in the measurement of the TG levels and total liver lipids content (Fig. 5H,I, respectively).

### CpKO mice overweight is not the result of an increase in food intake

**Fig. 6:**
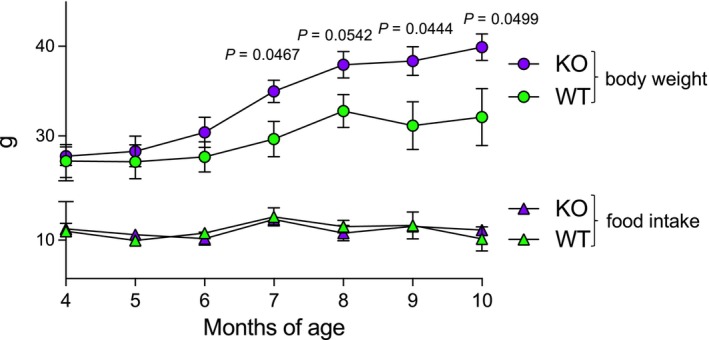
CpKO mice overweight is not the result of an increase in food intake. Longitudinal evaluation of body weight increase (upper part, dots) and food intake assessed over 3 days (lower part, triangles) in CpKO and WT mice (*n* = 6 mice per group) along aging from 4 to 10 months of age. Data are reported as the mean ± SEM of the animal groups; *P* values were evaluated by Student's *t* test comparing the two groups of animals at the same age.

Because we found significant accumulation of ω‐3 fas in the liver of CpKO mice compared to WT mice at 10 months, and because in mammals these lipids can be only obtained through the diet, we measured food intake in the two groups of animals along with aging in association with body weight increase. We confirmed in new groups of mice (*n* = 6 each group, three males and three females) that, until 6 months, no difference in body weight was evident in CpKO and WT mice, and that, from month 7 to month 10, CpKO mice were significantly overweight compared to WT mice (Fig. 6). This body weight increase was not associated with a parallel increase in food intake in CpKO mice because CpKO and WT mice ate similar amounts of food at all ages tested (CpKO 11.33 g and WT 11.27 g over 3 days on average per mouse) (Fig. 6). Similar food intake in CpKO and WT mice was confirmed in selected group of mice of 6 and 10 months of age where the analysis was performed considering the light and dark cycles over a period of 3 days (Fig. S4). Taken together, these results suggested a different lipid metabolism/catabolism in the two group of mice.

### CpKO mice show hepatic iron accumulation already at 6 months of age

**Fig. 7:**
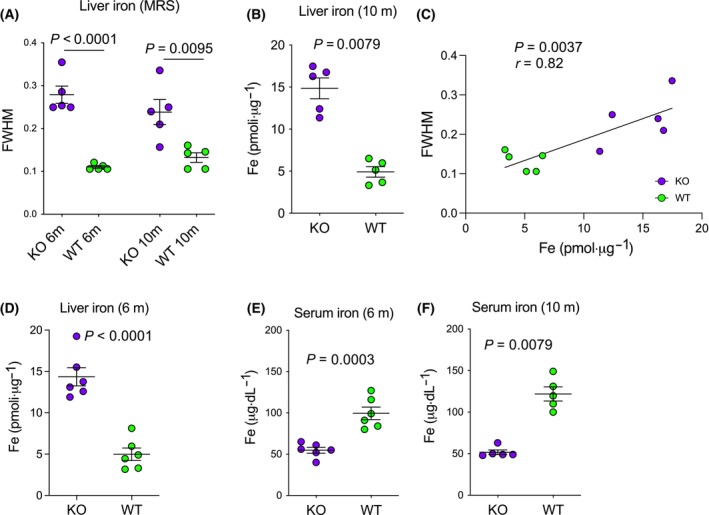
CpKO mice show liver iron accumulation at 6 and 10 months of age. (A) Liver iron level indirectly measured by ^1^H‐MRS *in vivo* in the mice at 6 and 10 months of age as water frequency width at half magnitude (FWHM) parameter. (B) *Ex vivo* evaluation by biochemical assay of iron level in the liver extracts of mice at 10 months of age. (C) Correlation analysis of the liver iron evaluation obtained by biochemical assay versus MRS quantitation. (D) Evaluation by biochemical assay of iron level in the liver extracts of mice at 6 months of age. (E, F) Evaluation by biochemical assay of iron level in the serum of mice at 6 and 10 months of age (E and F, respectively). In (A), (B), (D), (E) and (F), data are reported as the mean ± SEM of animal groups, with each dot corresponding to one mouse; *P* values were evaluated by Student's *t* test. In (C), each dot corresponds to one mouse; *P* values were evaluated by Pearson's test and the linear correlation coefficient *r* is reported.

We next, acquired information on iron content in the liver by evaluating the T2 relaxation value of the water peak parameter [water‐frequency width at half magnitude (FWHM)] in ^1^H‐MRS spectra, which indirectly reflects the iron abundance [36, 37]. Liver iron accumulation was measured as water FWHM *in vivo* in the mice at 6 and 10 months of age. A significant accumulation was found in CpKO mice already at 6 months compared to WT mice and this was confirmed in animals at 10 months (Fig. 7A). Iron deposition in the liver of the CpKO mice at 10 months was confirmed by biochemical evaluation performed *ex vivo* on tissue extracts at the end of the MRI analysis (Fig. 7B). The biochemical iron levels strongly correlate with the water FWHM measurements performed in the same animals (Fig. 7C). Iron accumulation in the liver of 6 months old CpKO mice was confirmed by iron biochemical evaluation in tissue extracts obtained from a different group of animals (Fig. 7D). Moreover, as expected for Cp‐deficient mice, liver iron accumulation was inversely correlated with serum iron level, which was lower in CpKO than in WT mice both at 6 and 10 months (54.8 and 51.8 μg·dL^−1^, respectively) and below the physiological range (80–170 μg·dL^−1^) (Fig. 7E,F).

## Discussion

In the present study, we used different methodological and analytical approaches to investigate, both *in vivo* and *ex vivo*, the amount and composition of lipids in the AT and liver of Cp‐deficient mice. *In vivo* MRI is widely used to investigate longitudinal body fat distribution in human and animal models [38, 39, 40, 41], whereas ^1^H‐MRS is used to assess, both non‐invasively and *in vivo*, the lipid composition in AT, visualizing the relevant water and lipid spectra [42]. In particular, ^1^H‐MRS has been successful to investigate hepatic TG and FA composition in several animal models [26, 27, 28, 43, 44]. Moreover, ^1^H‐MRS is also useful to concomitantly detect abnormal iron deposition in liver because it occurs in several metabolic diseases in which the accumulation of lipids is associated with iron deposition [42], as also observed in ACp [21, 23]. The liquid state ^1^H‐HR‐NMR is a quantitative and non‐destructive technique that enables an overview of the major lipid classes (fatty acids, glycerolipids, phospholipids and sterols) and some individual species in *ex vivo* biological samples [29]. ^1^H‐HR‐NMR, similar to ^1^H‐MRS, cannot provide a detailed characterization of the lipid species for the magnetically equivalent molecular structures of lipids that give largely overlapping resonances [30, 31]. To acquire precise quantitative data on lipids, it is essential to have 1H‐HR‐NMR standards. Because of this requirement, only a limited number of studies have been able to provide an absolute quantification of lipid metabolites in biological samples [30, 31]. In our research, we have utilized a in‐house lipids database for the quantification of lipids using ^1^H‐HR‐NMR.

Our results indicated that both CpKO and WT mice displayed a trend for accumulation of lipids during aging. However, the alteration of lipid metabolism in the CpKO mice is already present at 6 months of age, when differences in body weight are not yet significant. This is suggestive of a sort of accelerated metabolic aging in the Cp‐deficient mice compared to WT mice, as was similarly described for other chronic metabolic disorders, such as obesity and type 2 diabetes development [45]. In these pathologies, a feature of the aging process is the dysfunctionality of the AT that fosters systemic metabolic alterations, such as insulin resistance, accumulation of ectopic lipids and chronic inflammation [45]. Interestingly, diabetes is a hallmark of ACp patients who, in some cases, also show lipid metabolism dysregulation (i.e. overweight, obesity, liver steatosis and high cholesterol) [10, 21, 22, 24]. Notably, some of these characteristics are also displayed by Cp‐deficient mice [15, 23, 46]. Our results indicated signs of metabolic alteration in the liver of 6‐month‐old CpKO mice. This alteration appears to follow a precise order of sequential events, namely iron deposition, which promotes tissue inflammation, as witnessed by macrophage infiltration, followed by lipid deposition, as shown by the content of total hepatic lipids and the trend for an increase in accumulation of lipid macrovesicles, which is overt steatosis in some mice. By contrast, excluding the larger volume detected, AT did not display hypertrophy or inflammation in the CpKO mice at this age. The reason for AT accumulation in CpKO mice deserves further investigation. Therefore, the liver appears to play a pivotal role in the development of iron/lipid dysmetabolism in young CpKO mice. The dysmetabolism becomes overt in aged animals both in the liver, where it results in functional damage, and also in the AT, which shows hypertrophy and clustered macrophage infiltration.

Important differences between CpKO and WT mice, as highlighted using the different approaches, also arise at 10 months of age. ^1^H‐HR‐NMR *ex vivo* analysis, which is more sensitive, results in a more resolved spectra than MRS and allows absolute lipid‐chemical group quantification to indicate a significantly higher deposition of total lipids, TG and FA in the liver of CpKO mice compared to WT mice. On the other hand, the MRS *in vivo* approach, which has the advantage of allowing longitudinal assessments in the same animal, allowed us to observe the switch from unsaturated to saturated FA occurring in the CpKO mice as associated with lipid deposition for storage in macrovesicles within the hepatocytes [44]. Conversely, WT mice increase the amount of unsaturated FA during aging without showing a switch to SFA. An increase in UFA contents is a transitory condition that has been reported to precede the shift from unsaturated to saturated FA, a necessary passage for ectopic lipid deposition in the liver [44]. Indeed, WT mice at 10 months of age showed an increase in UFA but did not yet show deposition of liver lipid macrovesicles. Within the hypothesis of accelerated metabolic changes in CpKO, it is conceivable that the transitory UFA increase and the shift to SFA occurred in these mice in a time frame between 6 and 10 months of age, and thus this was not recorded in our analysis performed in 10‐month‐old mice. Even if a physiological trend for an increase in lipid accumulation during aging was observable in both genotypes, the nature of the accumulated lipids differed, consistent with an anticipation of the metabolic changes of aging in CpKO mice.

The ^1^H‐HR‐NMR analysis also revealed accumulation of the ω‐3 fas in the liver of CpKO mice at 10 months, a feature not measurable with MRS because the ω‐3 fas are not or hardly detectable with this technique [27]. Because ω‐3 fas cannot be synthesized by mammals and are obtained through the diet, we hypothesized that there would be different feeding behaviors between the two groups of animals. Based on the reported anxiety status of CpKO mice [47], we hypothesized that they might exhibit increased food intake. However, we ruled out that the overweight of CpKO mice and AT accumulation were associated with a higher food intake. We thus concluded that the accumulation of ω‐3 fas in the liver of CpKO mice might be the result of an altered lipids metabolism/catabolism, in line with an increased synthesis or reduced degradation of FA. We cannot exclude that the different energy balance between the two genotypes might be explained by the different adsorption of diet components, or by genotype‐dependent variations in the excretion of different lipids. For example the altered iron availability typical of Cp‐deficient mice [1, 7, 8, 9, 12] might affect the gut microbiota environment, thus influencing diet components adsorption as reported previously [48]. This topic will be the subject of future investigations.

A reduction of serum Cp level in non‐alcoholic fatty liver disease (NAFLD) and non‐alcoholic steatohepatitis patients further supports the hypothesis that Cp might play a role in the liver/AT cross‐talk and in lipid homeostasis [49, 50, 51, 52]. In a cohort of hepatic‐steatosis patients, an inverse correlation between circulating Cp levels and liver fat‐accumulation has been reported measuring the liver lipids contents by MRS [53]. The modulation of Cp in the liver/AT cross‐talk is further underlined by the data obtained in a rat model of acute liver Cp deficiency in which adipocytes increase their Cp expression to compensate for the Cp circulating level and function [54]. Moreover, in WT mice, Cp expression level in the AT has been reported to be more than twice that of the liver, suggesting an important role for AT in Cp homeostasis [23]. In a mouse model of hepatocyte‐specific Cp depletion, the animals did not show iron accumulation in the liver [55], which instead is a hallmark of the global CpKO mouse model of ACp [9, 15, 20, 23]. This suggests that a reduction in circulating Cp levels, as consequence of limited secretion of Cp by other tissues rather than the liver (e.g. AT), is fundamental to fostering alterations in both iron and lipid metabolism. This may have implications in metabolic pathologies, such as NAFLD, that are characterized by aberrant iron/lipids homeostasis and reduced Cp levels [49, 50, 51, 52] [56]. Exploiting the water peak FWHM parameter of the MRS analysis, which is affected by the presence of iron and indirectly reflects its abundance [36, 37], we collected longitudinal information of iron accumulation in the liver of the animals. CpKO mice showed robust iron deposition already at 6 months, confirming *in vivo* the data obtained *ex vivo* and previously reported observations [12, 20]. A good correlation between MRS and biochemical iron measurement performed on the same animals was found at 10 months of age, supporting the reliability of both the results and the techniques. Similar conclusions were inferred by the correlation found for both TG levels and total lipid content measured by different approaches, further indicating that the results obtained were comparable.

In conclusion, the different methodological approaches used to investigate lipid dysmetabolism in the Cp‐deficient mice yielded complementary information. The combined use of these techniques constitutes an added value to follow disease progression: (a) it improves the phenotypic and biochemical assessment of metabolic diseases and (b) it allows to longitudinal analysis to be performed *in vivo*, highlighting the relevant changes associated with disorder evolution. This is particularly relevant in metabolic diseases where lipids and iron deposition occur concomitantly because these two parameters can be evaluated simultaneously with such a technique. The possibility to acquire a detailed account of disease progression has enabled us to highlight the significant impact of alterations in liver metabolism. This process commences with the accumulation of iron, subsequently leading to liver inflammation and the accumulation of lipids. Of note, these features became overt for AT only during advanced stages of the disease, again revealing the importance of longitudinal metabolic analysis. Finally, our data suggest that alterations in lipid metabolism play a role in the pathological mechanisms of ACp when coupled with disruptions in iron homeostasis.

## Conflicts of interest

MA discloses that the study was partially financial supported by Kedrion SpA within the frame of a scientific collaboration.

## Author contributions

MA conceived and coordinated the study and wrote the manuscript. LC and TC performed MRI and MRS analysis. VM and GM executed NMR analysis and contributed to manuscript editing. VM performed statistical analysis; AZ, SR, BF and ACo handled mice and performed biochemical experiments. ACa contributed to study design and manuscript editing. All the authors discussed the results and commented on the manuscript at all stages.

## Supporting information


**Fig. S1.** MRI analysis.
**Fig. S2.** Evaluation of liver size.
**Fig. S3.** Glucose tolerance test and insulin tolerance test.
**Fig. S4.** Food intake in light and dark cycles.
**Table S1.** Fatty acid composition indices generated from proton magnetic resonance spectra (^1^H‐MRS) of hepatic lipids.
**Table S2.** Lipid molecules from Merck/Sigma Aldrich used to build an ‘in‐house’ database for ^1^H‐HR‐NMR resonance.
**Table S3.** Measured parameters.

## Data Availability

The datasets are available from the San Raffaele Open Research Data Repository” (ORDR) (https://ordr.hsr.it/research‐data); doi: 10.17632/5skx2c69nr.1.
