# Supplementary material for: Lipid dysmetabolism in ceruloplasmin‐deficient mice revealed both in vivo and ex vivo by MRI, MRS and NMR analyses
Source: FEBS Open Bio. 2023 Dec 15;14(2):258–75. doi: 10.1002/2211-5463.13740 (PMC10839333; doi:10.1002/2211-5463.13740)
Supplement: Supplementary file 1 — Fig. S1. MRI analysis. Fig. S2. Evaluation of liver size. Fig. S3. Glucose tolerance test and insulin tolerance test. Fig. S4. Food intake in light and dark cycles. Table S1. Fatty acid composition indices generated from proton magnetic resonance spectra (1H‐MRS) of hepatic lipids. Table S2. Lipid molecules from Merck/Sigma Aldrich used to build an ‘in‐house’ database for 1H‐HR‐NMR resonance. Table S3. Measured parameters. [file FEB4-14-258-s001.pdf]

Supplementary Materials

Lipid dysmetabolism in ceruloplasmin-deficient mice revealed both *in vivo* and *ex vivo* by MRI, MRS and NMR analyses

Mannella V, Chaabane L, Canu T, Zanardi A, Raia S, Conti A, Ferrini B, Caricasole A, Musco G, Alessio M.

Inventory of Supplementary Materials:

Supplementary Figures S1 – S4

Supplementary Table S1 - S3

Supplementary Figure S1

<u>MRI analysis</u>. The analysis was performed using a 7-Tesla preclinical scanner (BioSpec 70/30 USR, Paravision 6.0.1, Bruker BioSpin, MRI GmbH, Ettligen, Germany) as reported in Materials and Methods.

A

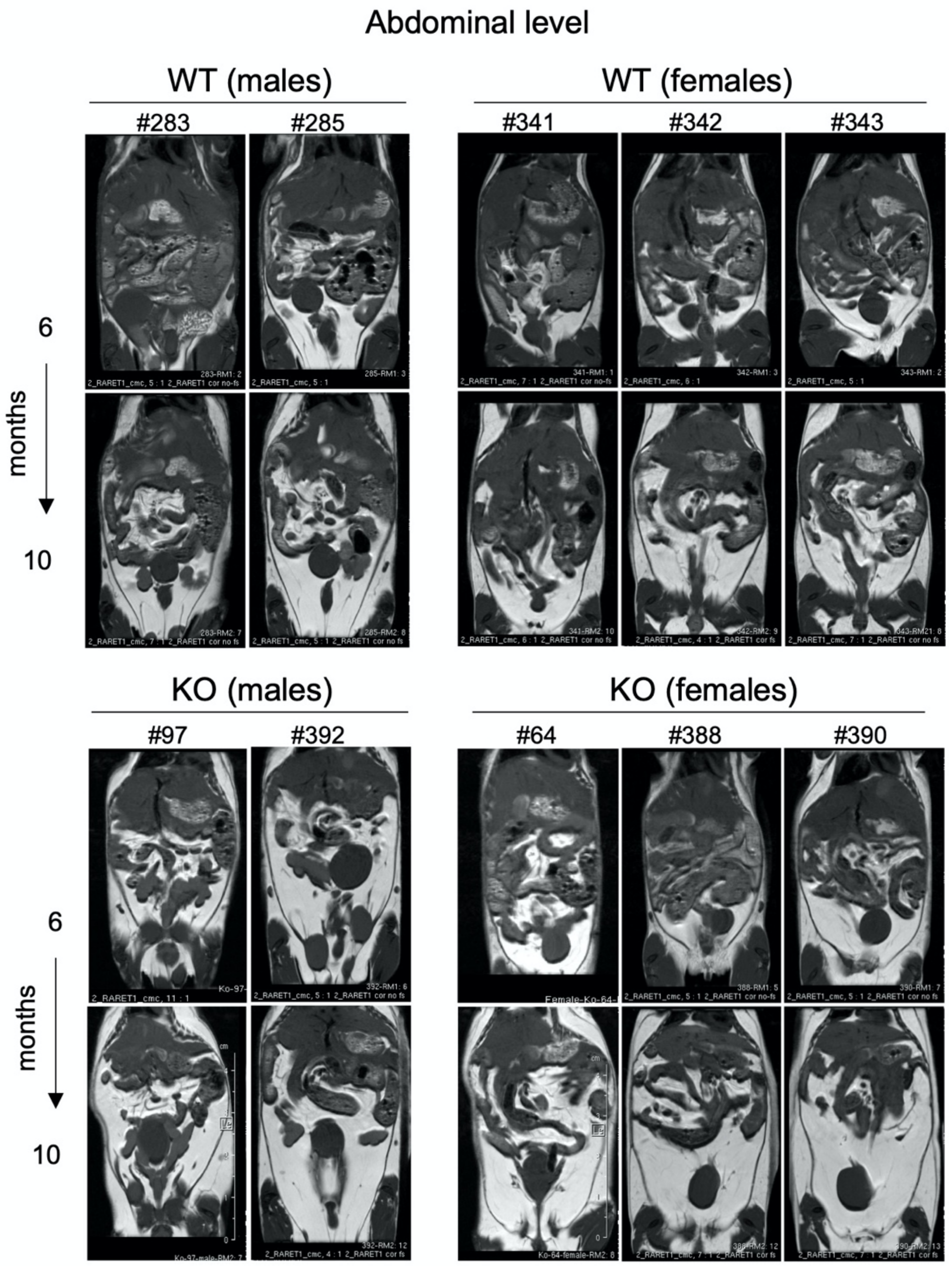

B

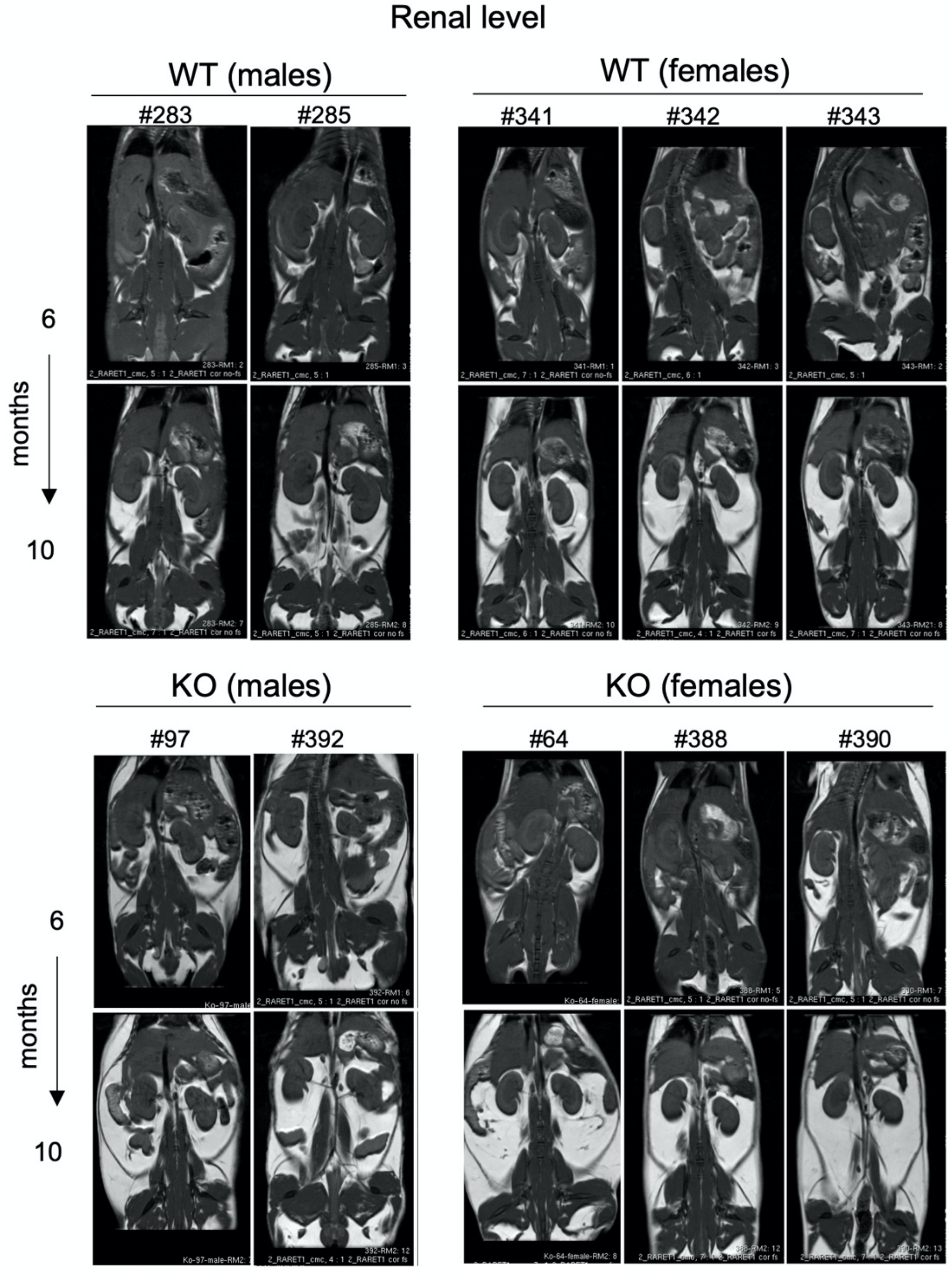

Figure S1: Representative sagittal 2D-high-resolution rapid acquisition with relaxation enhancement T1 images acquired without fat suppression at abdominal (**A**) and renal (**B**) levels, used for *in vivo* quantification of the adipose tissue volume of all CpKO and WT experimental mice at 6 and 10 months of age.

Supplementary Figure S2

Evaluation of liver size. The MRI analysis was performed using a 7-Tesla preclinical scanner as reported in Materials and Methods on both mice at 6 and 10 months of age. For liver volume quantification, manual segmentation was performed on each slice using the RARE-T2 sequence without fat suppression, and volume results from automatic summation of voxel volumes; images were analysed with MIPAV software. At 10 months of age mice were euthanized by transcardial perfusion with saline and organs were collected.

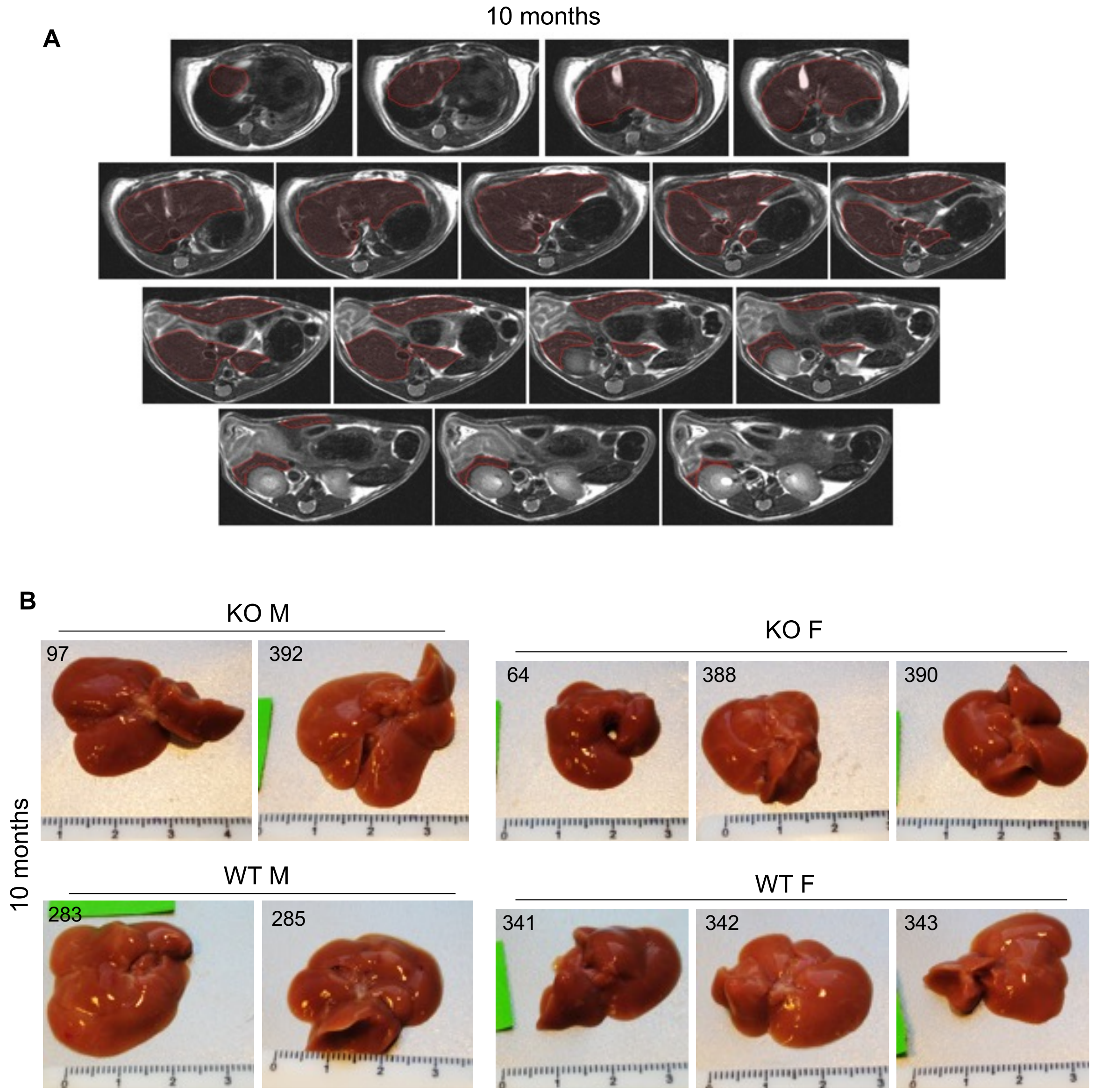

Figure S2: (**A**) Representative manual segmentation of liver performed on slices of the RARE-T2 sequence and used for liver volume reconstruction. For the value of each mouse see Table S3. (**B**) Liver of CpKO and WT mice at 10 months of age.

Supplementary Figure S3

<u>Glucose tolerance test (GTT) and insulin tolerance test (ITT)</u>. Tests were performed evaluating blood glucose level measured using a Contour Care glucometer (Ascensia Diabetes Care) as described in Materials and Methods in new groups of CpKO and WT mice at 6 and 10 months of age (groups “6 months bis” and “10 months bis”).

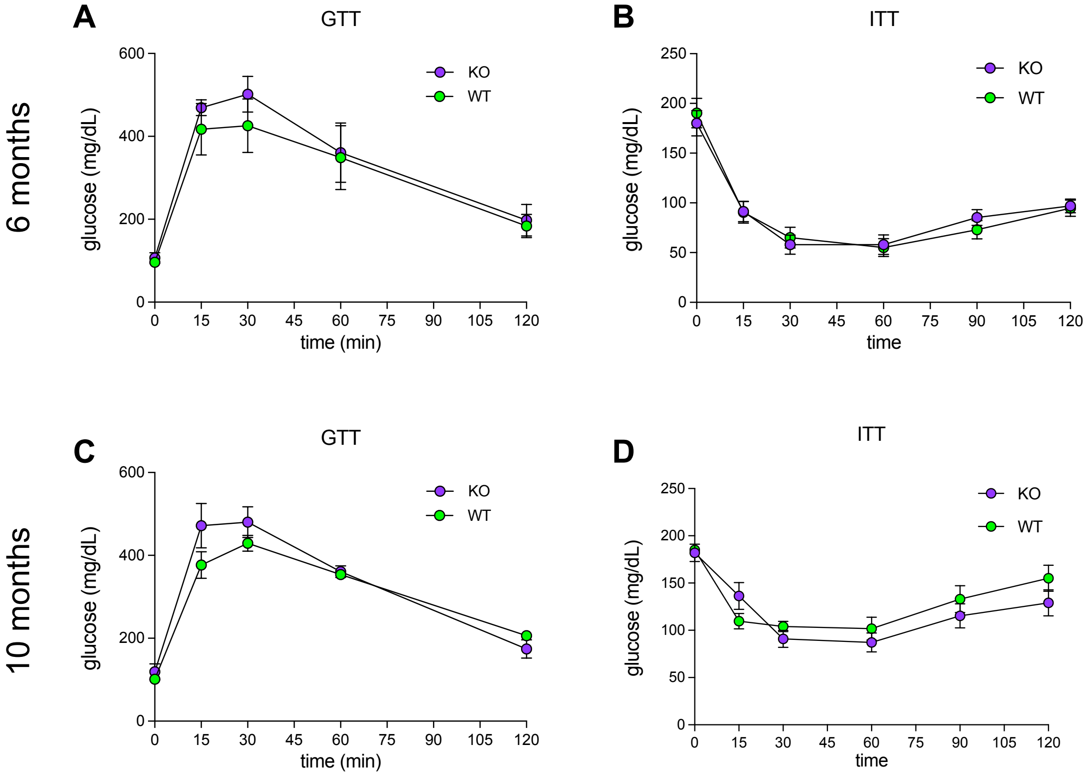

Figure S3: (**A** and **C**) Glucose and (**B** and **D**) insulin tolerance test (GTT and ITT respectively) performed on CpKO (purple) and WT (green) mice at 6 and 10 months of age (n= 6 mice/group, 3 males and 3 females). Data are shown as mean ± SEM of glucose concentration in the blood over 120 minutes from glucose or insulin administration. Statistical comparison of the two group of mice was done by Student’s t test performed on the different time points and as comparison of the areas under the curve (AUC). For the value of each mouse AUC see Table S3.

Supplementary Figure S4

Food intake in light and dark cycles. Food intake in light and dark cycle was evaluated in selected group of mice at 6 and 10 months of age every 12 hours for a total of 3 days, as described in Materials and Methods. Mice were weighted to confirm CpKO overweight at 10 months of age.

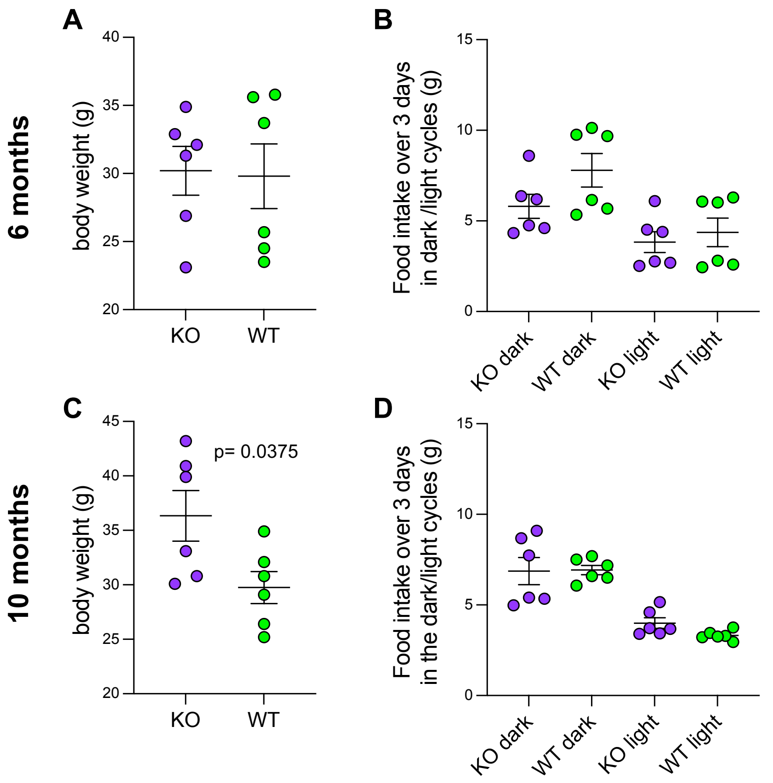

Figure S4: (**A** and **C**) Body weight evaluation of CpKO and WT mice at 6 and 10 months of age, respectively. (**B** and **D**) Evaluation of food intake in the 12 hours dark/light cycles assessed over 3 days and reported as grams of aet food at 6 and 10 months of age. Data are reported as mean ± SEM of the animal groups, each dot corresponds to one animal (n= 6 mice/group, 3 males and 3 females); statistical p-values were evaluated by Student's t test comparing the two groups of animals at the same age and same phase of the cycle. For the value of each mouse see Table S3.

Supplementary Table S1

Fatty acid composition indices generated from proton magnetic resonance spectra (1H-MRS) of hepatic lipids	
Saturation index (SI)	1.3 ppm x 3 / 0.9 ppm x 2
Number of double bonds (ndb)	5.2+5.3 ppm x 3 / 0.9 ppm x 2
Unsaturated fatty acids (UFA)	2.1 ppm x 3 / 0.9 ppm x 4
Saturated fatty acids (SFA)	1 - UFA
Polyunsaturated fatty acids (PUFA)	2.8 ppm x 3 / 0.9 ppm x 2
Monounsaturated fatty acids (MUFA)	UFA - PUFA
Mean chain length (MCL)	[0.5 x (1.3 ppm + 2.1 ppm + 2.8 ppm) + (5.2 ppm + 5.3 ppm)] / 0.33 x 0.9 ppm

Chemically distinct fatty-acyl protons are identified by their chemical shift in the 1H-MRS as indicated in Figure 3B in the text

Indices were defined accordig to Soares *et al.* (NMR Biomed 2015;28:1009–20)

Supplementary Table S2

Lipid molecules from Merck/Sigma Aldrich used to build an “in-house” database for 1H-HR-NMR resonance

Product code	Lipid name	Lipids classes
22244-25MG	CERAMIDE	CERAMIDES
P7943-5MG	L-ALPHA-PHOSPHATIDYLETHANOLAMINE	PHOSPHATIDYLETHANOLAMINE
P9511-5MG	3-SN-PHOSPHADITIC ACID SODIUM SALT	GLYCEROPHOSPHOLIPIDS
50445-10MG	1-(3-SN-PHOSPHATIDYL)-RAC-GLYCEROL SODIUM SALT	
79403-5MG	PHOSPHATIDYLINOSITOL SODIUM SALT	
855375P-25MG	10:0 LYSO PHOSPHOCHOLINE	LYSOPHOSPHOLIPIDS
P3556-100MG	L-A-PHOSPHATIDYLCHOLINE	PHOSPHATIDYLCHOLINE
P7769-25MG	1,2-DIACYL-SN-GLYCERO-3-PHOSPHO-L-SERINE	GLYCEROLPHOSPHOSERINE
S0756-50MG	SPHINGOMYELIN	SPHINGOLIPIDS
1787-1AMP	MONO-, DI- & TRIGLYCERIDE MIX	TRIGLYCERIDES
C8667-500MG	CHOLESTEROL SIGMA GRADE	STEROLS
C6072-1G	CHOLESTERYL PALMITATE 98%	
UN10-1KT	PALMITOLEIC ACID	UNSATURATED FATTY ACIDS
UN10-1KT	OLEIC ACID	
UN10-1KT	LINOLEIC ACID	
UN10-1KT	ARACHIDONIC ACID	
UN10-1KT	LINOLEINIC ACID	
EC10A-1KT	PALMITIC ACID	SATURATED FATTY ACIDS
EC10A-1KT	STEARIC ACID	
EC10A-1KT	ARACHIDIC ACID	
EC10A-1KT	LAURIC ACID	

Supplementary Table S3

Supplemental Materials SM-Table 3. Measured parmeters

mouse	sex	Lenght 6 m (cm)	Lenght 10 m (cm)	Weight 6 m (g)	Weight 10 m (g)	MRI adipose tissue: Volume 6 m (mm^3)	MRI adipose tissue: Volume 10 m (mm^3)	MRI liver: Volume 6 m (mm^3)	MRI liver: Volume 10 m (mm^3)	NMR adipose tissue (10 m): Total lipid (area spectra/mg)	TG (mM/mg FW)	PC+LPC (mM/mg FW)	PE (mM/mg FW)	ω-3 Fas (mM/mg FW)	FA (mM/mg FW)	TC (mM/mg FW)	MRS liver (6 m): IHLC	SI	ndb	UFA	SFA	PUFA	MUFA	MCL	TG	Water FWHM
KO97	m	7.0	7.0	36.80	41.80	4619.5	5893.2	1258	1522	430691	0.6757	0.00152	0.00019	0.0369	1.5584	0.00247	0.102	2.272	0.280	0.79	0.21	1.509	0.001	9.85	0.00237	0.250
KO392	m	7.3	7.2	34.00	38.90	4058.9	4471.5	1036	1364	570269	0.8943	0.00182	0.00019	0.0514	2.0654	0.00326	0.049	8.858	0.880	0.67	0.33	0.530	0.14	16.25	0.00057	0.355
KO64	f	6.7	6.9	31.50	41.53	2995.5	7421.2	865	1114	551147	0.8653	0.00141	0.00017	0.0658	1.9832	0.00238	0.108	13.265	1.010	0.89	0.11	0.300	0.59	21.10	0.00139	0.250
KO388	f	6.7	7.2	23.80	31.30	1482.9	6422.0	713	1083	431840	0.6834	0.00062	0.00488	0.0563	1.5911	0.00269	0.055	14.707	0.090	0.84	0.16	0.530	0.31	21.07	0.00028	0.286
KO390	f	6.9	7.4	26.40	38.50	3741.6	8706.2	783	1271	476513	0.7693	0.00058	0.00413	0.0613	1.7415	0.00214	0.097	12.769	0.740	1.03	0.001	0.590	0.44	20.71	0.00071	0.254
WT283	m	7.0	7.3	34.26	33.55	770.7	2665.5	1939	1274	558254	0.8855	0.00104	0.00637	0.0678	2.0271	0.00244	0.014	7.679	0.050	0.42	0.58	0.580	0.42	12.61	0.00008	0.113
WT285	m	7.2	7.4	33.01	37.56	1666.4	3881.0	1339	1528	446262	0.7004	0.00026	0.00027	0.0468	1.6129	0.00243	0.023	7.279	0.130	0.43	0.57	0.570	0.43	12.36	0.00020	0.121
WT341	f	6.5	6.9	25.43	29.85	1705.0	4040.3	823	1025	503697	0.8045	0.00085	0.00560	0.0619	1.8489	0.00250	0.053	13.275	0.110	0.87	0.13	0.390	0.49	19.60	0.00028	0.106
WT342	f	6.3	7.3	24.40	30.35	1712.6	4428.5	731	850	484209	0.7635	0.00076	0.00345	0.0430	1.7517	0.00209	0.044	10.852	0.750	0.97	0.03	0.500	0.47	18.61	0.00032	0.106
WT343	f	6.1	6.8	23.32	28.90	1796.8	2932.4	651	852	582706	0.9203	0.00109	0.00522	0.0620	2.1340	0.00353	0.071	10.313	1.300	0.76	0.24	0.540	0.22	18.65	0.00050	0.106

mouse	MRS liver (10 m): IHLC	SI	ndb	UFA	SFA	PUFA	MUFA	MCL	TG	Water FWHM	NMR liver (10 m): HLC (area spectra/mg)	TG (mM/mg DW)	PC+LPC (mM/mg DW)	PE (mM/mg DW)	ω-3 Fas (mM/mg DW)	FA (mM/mg DW)	TC (mM/mg DW)	liver (10 m): Lipid vesicles (% area)	TG (µg/mg tissue)	Iron (pmol/µg prot)	F4/80 staining (% area)	serum (10 m): Iron (µg/dL)	ALT (U/L)	AST (U/L)	Adipose tissue (10 m): Adipocytes area (pixel)	Adipocytes diameter (pixel)	F4/80 staining (% cells)
KO97	0.138	9.349	1.444	0.635	0.365	0.167	0.384	17.38	0.00107	0.250	191013	0.2015	0.0041	0.0202	0.0485	0.7447	0.0139	4.79	17.37	12.41	12.65	63	44	96	141409	460	62.32
KO392	0.092	10.439	0.980	0.60	0.400	0.260	0.210	17.74	0.00073	0.157	158522	0.1752	0.0044	0.0299	0.0333	0.5281	0.0126	7.81	12.59	11.35	8.62	49	42	85	118674	419	38.79
KO64	0.139	13.885	0.820	0.89	0.001	0.330	0.818	22.44	0.00090	0.336	221847	0.2033	0.0065	0.0288	0.0462	0.6514	0.0253	6.27	24.62	17.47	8.55	50	138	204	94672	381	42.55
KO388	0.273	7.290	0.390	0.48	0.520	0.090	0.350	13.06	0.00171	0.210	186154	0.2017	0.0007	0.0252	0.0476	0.6787	0.0196	5.55	31.49	16.75	9.00	48	41	97	72049	331	51.23
KO390	0.127	1.355	0.080	0.31	0.690	0.290	0.001	6.54	0.00310	0.240	190799	0.2236	0.0022	0.0306	0.0487	0.6052	0.0216	7.40	26.83	16.28	11.73	49	67	111	63509	306	41.94
WT283	0.046	6.682	0.340	1.38	0.001	1.110	0.001	15.70	0.00067	0.161	130041	0.1151	0.0060	0.0276	0.0280	0.5241	0.0156	0.51	7.819	3.33	5.80	100	31	74	56086	284	22.87
WT285	0.102	8.937	0.150	0.57	0.430	0.270	0.163	14.74	0.00095	0.143	167696	0.1681	0.0169	0.0657	0.0421	0.5400	0.0128	1.33	15.93	3.67	7.62	149	27	109	51449	281	27.56
WT341	0.094	12.316	0.760	1.14	0.130	0.080	1.010	20.03	0.00077	0.106	172420	0.1860	0.0023	0.0348	0.0359	0.6113	0.0223	2.28	15.78	5.97	6.92	131	37	79	37254	237	38.84
WT342	0.128	12.946	0.690	1.07	0.030	0.190	0.777	20.58	0.00125	0.106	177340	0.1748	0.0043	0.0301	0.0379	0.5640	0.0175	3.11	19.24	5.14	6.05	110	36	101	42989	251	28.01
WT343	0.138	11.617	2.480	1.05	0.240	0.320	0.570	22.52	0.00073	0.146	138982	0.1456	0.0004	0.0202	0.0354	0.4770	0.0154	2.38	20.56	6.5	5.65	119.8	35.8	95.6	18342	158	29.42

6 months bis			liver (6 m)			serum (6 m)					Adipose tissue (6 m)			Food Intake (6 m)	
mouse	sex	Weight (g)	Lipid vesicles (% area)	Iron (pmol/µg prot)	F4/80 staining (% area)	Iron (µg/dL)	ALT (U/L)	AST (U/L)	GTT (AUC)	ITT (AUC)	Adipocytes area (pixel)	Adipocytes diameter (pixel)	F4/80 staining (% cells)	Dark (g/3 cycles)	Light (g/3 cycles)
KO1179	m	32.1	0.63	12.59	7.2	61	37	112	35543	5625	94666	362	53.14	6.19	4.39
KO1187	m	32.9	1.67	19.26	10.01	55	33	103	46725	9023	29097	205	40.52	8.59	6.09
KO1192	m	34.9	1.12	13.11	9.21	65	40	109	46163	1501	43942	261	24.61	6.37	4.52
KO1183	f	26.9	5.37	11.91	9.01	56	29	61	20648	7418	63950	307	28.94	4.33	2.52
KO1184	f	23.1	2.20	13.76	8.44	52	42	56	21818	5153	63210	316	19.41	4.75	2.77
KO1190	f	31.3	6.27	15.52	8.16	40	29	62	19328	5888	47425	268	35.72	4.61	2.69
WT1156	m	35.8	0.40	3.17	3.08	116	34	87	47505	8685	95972	366	31.23	9.68	6.02
WT1159	m	35.6	1.31	4.9	5.29	91	47	75	10838	5340	43688	248	31.07	9.76	6.07
WT1161	m	33.7	0.53	3.31	5.54	80	39	54	47528	3450	55030	285	27.25	10.13	6.3
WT1157	f	24.5	1.49	5.96	7.27	84	43	99	25568	5288	54072	279	12.32	5.35	2.44
WT1160	f	25.7	0.96	4.46	8.1	99	39	101	14010	8460	33737	224	22.51	5.68	2.59
WT1163	f	23.5	0.38	8.12	7.72	127	34	63	23505	1170	25031	188	21.59	6.16	2.81

10 months bis			Serum (10 m)		Food Intake (10 m)	
mouse	sex	Weight (g)	GTT (AUC)	ITT (AUC)	Dark (g/3 cycles)	Light (g/3 cycles)
KO938	m	40.9	27301	10425	8.68	5.16
KO959	m	39.9	32415	15563	9.09	3.4
KO1004	m	43.2	25350	9810	7.73	4.59
KO928	f	33.1	31628	7920	5.4	3.72
KO943	f	30.1	31943	9120	4.98	3.43
KO940	f	30.8	35168	6383	5.34	3.68
WT996	m	34.9	26738	12068	7.18	3.22
WT997	m	29.1	24765	6563	6.6	2.96
WT998	m	32.1	30045	9923	7.69	3.45
WT1000	f	26.4	32505	12518	6.07	3.29
WT1001	f	25.2	28185	11805	7.51	3.76
WT1003	f	30.8	29115	13965	6.5	3.25
